# Impact of smoking on the complement system: a narrative review

**DOI:** 10.3389/fimmu.2025.1619835

**Published:** 2025-06-19

**Authors:** Ahmed B. Alarabi, Fatima Z. Alshbool, Fadi T. Khasawneh

**Affiliations:** ^1^ Department of Pharmaceutical Sciences, College of Pharmacy, Texas A&M University, Kingsville, TX, United States; ^2^ Department of Pharmacy Practice, College of Pharmacy, Texas A&M University, Kingsville, TX, United States

**Keywords:** smoking, complement system, inflammation, cardiovascular disease, lung disease, e-cigarette, tobacco glycoprotein, particulate matter

## Abstract

Smoking is a major cause of morbidity and mortality, resulting in an increased risk of cardiovascular, respiratory, inflammatory, and degenerative diseases. In this review, we highlight the complex interactions between smoking and activation of different components of the complement system, in order to underscore the notion that its dysregulation underlies—mechanistically—as well as exacerbates the progression of a host of disease processes. Moreover, we also briefly delve into components of tobacco smoke—including chemical constituents like tobacco glycoprotein (TGP) and particulate matter (PM), toxic metals, and other mainstream cigarette smoke chemicals—that have been identified as possible culprits in complement activation. In doing so, this review makes important and meaningful contributions to the ongoing efforts of combating the global health crisis posed by tobacco use, all while emphasizing the need for multifaceted strategies that include not only public health measures and educational efforts, but also innovative research that focuses on understanding and mitigating the biological mechanisms underlying smoking-related health conditions.

## Introduction

While data show a declining trend, tobacco use remains a leading cause of preventable diseases, disabilities, and deaths in the U.S., as per the Centers for Disease Control and Prevention (CDC). Indeed, around 28.3 million adults and 2.8 million middle and high school students in the U.S. use tobacco products (1). Smoking and secondhand smoke exposure lead to nearly 500,000 premature deaths annually, while 16 million Americans suffer from serious smoking-related illnesses. In terms of the economic impact, the U.S. incurs over $225 billion in annual medical expenses to address diseases caused by smoking (1). While these numbers reflect a persistent public health challenge, they also underscore the critical need for effective strategies to further reduce tobacco use as well as mitigate its profound and detrimental impact on health. Consequently, exploring the multifaceted mechanisms of how smoking can participate in disease pathogenesis is essential for highlighting evidence to educate the public, and for developing targeted approaches to reduce its impact on health.

There is ample evidence that links smoking to several health issues that span virtually every system within the human body, which underscores its role as a primary contributor to morbidity and mortality not only in the U.S. but worldwide. To this end, cardiovascular diseases, including heart attacks and stroke, are significantly more common among smokers (2), as are a variety of cancers (3). Respiratory diseases, such as chronic obstructive pulmonary disease (COPD) and emphysema, are markedly exacerbated by smoking, which also compromises the body’s ability to fight infections, leading to increased susceptibility to pneumonia and tuberculosis (4). Smoking was also found to impact reproductive health, thereby contributing to fertility problems (5), as well as pregnancy complications (6), and/or even adverse outcomes for the offspring due to maternal exposures (7). This broad spectrum of smoking-related disorders not only reflects the significant and extensive impact tobacco use exerts on human health, but also highlights the critical need for a better/further understanding of the underlying pathological processes.

The detrimental health effects of smoking can be attributed to a complex interplay of ‘harmful’ mechanisms that are triggered by more than 7,000 chemicals known to be present in tobacco smoke, of which hundreds are toxic and at least 69 are known carcinogens (8, 9). To this end, these chemicals have been found to produce a host of effects such as initiate oxidative stress, promote inflammation, induce DNA damage, and impair cellular and systemic immune responses, collectively contributing to the development and progression of various diseases (9). Among the less explored yet pivotal pathways affected by smoking is the complement system—which is a key component of the immune system that enhances (aka complements) the ability of antibodies and phagocytic cells to clear pathogenic invaders (10). While the complement system plays a crucial role in defending against infection and disease, its aberrant activation by smoking could in fact lead to excessive inflammation, tissue damage, and a cascade of immune responses that contribute to the pathogenesis of smoking-related diseases (11, 12). In fact, complement system activation has been linked to a number of major chronic disease conditions including cancer, cardiovascular, autoimmune, and renal diseases (13–15). Furthermore, therapeutic targeting of the complement system components yielded very promising results in the treatment of different disease conditions, which is exhaustively reviewed in (16). Therefore, summarizing what is known about the interplay between smoking and the complement system should provide potential/crucial insight that is currently lacking from a clinical and research stand points, thereby paving the way for researchers to focus on this important system; and also for guiding the development of targeted therapeutic strategies and interventions designed to ameliorate the related adverse health effects associated with tobacco use.

In summary, by examining the intricate ways in which smoking influences the complement system, which, as indicated before, is a fundamental aspect of the immune response, we aim to provide a fresh perspective on the pathophysiological processes underlying smoking-related health conditions. And in doing so, this review contributes to a more comprehensive approach to combating the global health crisis posed by tobacco use, while emphasizing the need for multifaceted strategies that include not only public health measures and educational efforts but also innovative research focused on understanding and mitigating the biological mechanisms through which smoking inflicts its harm.

## Overview of the complement system

The complement system is a critical component of the immune system, serving as a bridge between innate and adaptive immunity. It comprises of over 50 glycoproteins, both soluble and membrane-bound, that work together in a complex network of protein-protein interactions. This network functions to recognize and eliminate pathogens through a series of proteolytic reactions that result in key immune processes, including opsonization of pathogens, recruitment of inflammatory cells via anaphylatoxins (e.g., C3a, C5a), and direct lysis of target cells through the formation of the membrane attack complex (MAC) (17, 18).

**Figure 1:**
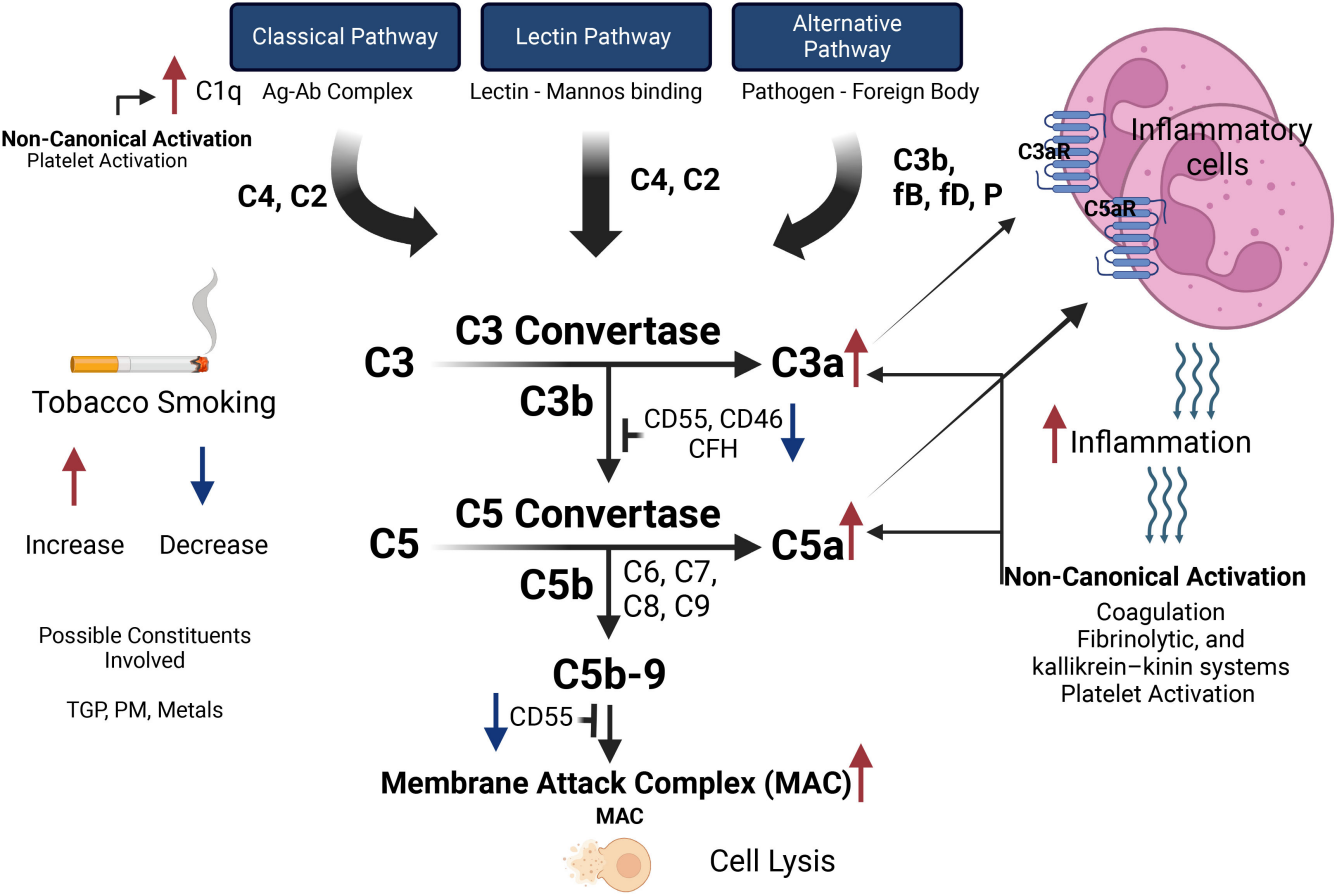
The effects of tobacco smoking on the activation pathways of the complement system and subsequent inflammatory responses. The complement system can be activated via the Classical Pathway (initiated by the antigen-antibody complex, activating C1q), the Lectin Pathway (triggered by lectin binding to mannose), and the Alternative Pathway (activated by pathogens or foreign bodies, involving C3b, factor B, factor D, and properdin). These pathways converge at C3 convertase, which cleaves C3 into C3a (promoting inflammation via C3a receptor) and C3b (enhancing opsonization). C5 convertase then cleaves C5 into C5a (recruiting inflammatory cells via C5a receptor) and C5b (initiating the formation of the Membrane Attack Complex leading to cell lysis). Tobacco smoking increases C1q levels, enhancing Classical Pathway activation, while constituents like tobacco glycoprotein, particulate matter, and metals influence complement activity. Smoking decreases regulatory proteins (CD55, CD46, CFH), increasing complement activation, and elevates C3a and C5a levels, heightening inflammation and cell recruitment. Non-canonical pathways such as coagulation, fibrinolytic, and kallikrein-kinin systems, and platelet activation can be triggered by inflammatory response and participate in activating the complement system.

There are three canonical pathways that are known to initiate the activation of the complement system, those are: 1. classical, 2. alternative, and 3. lectin pathways (18), each of which is triggered/activated by different signals. To this end, the classical pathway begins with the attachment of the C1q molecule either to antibodies that are bound to pathogens, or directly to pathogens. C1q is part of the C1 complex, which comprises a single C1q molecule linked to two molecules each, namely C1r and C1s. Once activated (C1s), it acts on the next two components of the classical pathway, cleaving C4 and then C2 to generate two large fragments, C4b and C2b, which together form the C3 convertase of the classical pathway (**Figure 1**). As for the lectin pathway, it is initiated by the binding of pattern-recognition molecules like mannose-binding lectin (MBL) to specific sugars on the surface of microorganisms or damaged host cells. When the MBL complex- whose components are MASP-1 and MASP-2- binds to a pathogen surface, this leads to cleavage of C4 and C2. Finally, the alternative pathway is continuously active at low levels due to the spontaneous hydrolysis of C3, a condition known as “C3 tick over” (19). This pathway can quickly amplify the immune response once a pathogen or other non-pathogenic invaders are detected, thereby leading to the generation of a distinct C3 convertase, namely C3bBb (18, 20).

At the heart of the action of the complement system is the cleavage of the C3 protein into C3b and C3a by the C3 convertases formed by the trigger of the canonical pathways (classical, alternative, and lectin). C3b then tags pathogens for destruction through a process called opsonization, making them easier targets for immune cell phagocytosis. C3a, along with another fragment called C5a that is generated from the cleavage of C5 by C5 convertases, plays a significant role in inflammation and immune modulation by robustly attracting inflammatory cells (C3a and C5a are also called anaphylotoxins) (18). It is important to note that the complement system is finely tuned to prevent excessive activation and damage to host tissues. Indeed, there are various regulatory proteins (**Table 1**) that ensure that complement activity is precisely controlled, such that there is balancing of the need for a rapid response to invaders all while preserving/protecting the host’s cells (30).

**Table 1:** Complement System Regulators.

Complement system regulators	Type	Mechanism of regulation
Factor H and related proteins	Fluid-phase	Facilitates the decay of C3 convertases, acts as a cofactor for inactivation of C3b, and recognizes host surfaces to prevent self-attack (18).
Factor I (CFI)	Fluid-phase	Serine protease that cleaves and inactivates C3b and C4b in the presence of cofactors such as Factor H, MCP (CD46), or C4BP (21).
C4b-binding protein (C4BP)	Fluid-phase	Acts as a cofactor for inactivation of C4b, playing a role in limiting classical and lectin pathway activations (22). Decay-accelerating activity for the classical C3 convertase and as a cofactor for the cleavage of C3b (23, 24).
CD46 (MCP)	Membrane-bound	Regulate complement activation on cell surfaces by acting as cofactors (CFI) for the inactivation of C3b and C4b (25, 26)
Decay-Accelerating Factor (DAF)	Membrane-bound	Accelerates the decay of C3 and C5 convertases, preventing the amplification of complement responses on host cells (26).
CD59	Membrane-bound	Prevents the formation of the MAC by inhibiting the assembly of C5b-9 complexes, protecting host cells from lysis (27).
Carboxypeptidases N, R, B2	Fluid-phase	Regulate magnitude of complement activation of C3a and C5a, reducing their inflammatory potential (28).
C1-esterase inhibitor	Fluid-phase	Inhibits the classical and lectin pathway proteases (C1r, C1s, MASPs), controlling the activation of these pathways (29).

In addition to the canonical pathways of activation, the complement system can also be activated through non-canonical routes. To this end, enzymatic activity from systems such as coagulation has been shown to activate the complement system, which was illustrated to be mainly due to the ability of FXa, FXIa and plasmin to cleave both C5 and C3, and robustly generate C5a and C3a, with the latter being proinflammatory molecules (31). It was also shown that thrombin can cleave C5, especially in the absence of C3 substituting for the C3-dependent C5 convertase (32) a process that confirms a close and intertwined relationship between coagulation and the complement system. Other non-canonical pathways such as fibrinolysis, and kallikrein–kinin systems can directly cleave complement proteins, bypassing the need for convertases (33, 34). It was also shown that platelets can contribute to activating the complement’s classic pathway through the paracrine complement activating mediator chondroitin sulfate A, which is expressed on the platelet surface upon activation and binds C1q along with many other complement components (35). It was also reported that microparticles released from activated platelets can participate in activating the complement system (36). In addition, thrombin-activated platelets were shown to initiate the alternative complement cascade (37) whereas the platelet p-selectin was found to independently activate, at least, parts of the complement components (38). These non-canonical pathways may play roles in various conditions, including inflammatory diseases and thrombo-inflammation, both of which are important contributors to the pathogenesis of a host of different diseases states. It is also worth mentioning that the complement system in and of itself is increasingly recognized for its non-canonical roles, which go beyond the innate immunity domain. These include regulating neuronal development, tissue repair, and metabolic homeostasis (39, 40). Indeed, there is evidence showing that complement system activity also operates intracellularly, where it interacts with innate sensors such as the pyrin domains-containing protein 3 (NLRP3) inflammasome and mitochondrial anti-viral signaling protein (MAVS), to coordinate responses to cellular stress and danger signals (41). However, this area is still under active investigation and more work is needed to solidify this non canonical role of the complement system in the development of disease conditions.

Understanding the fundamental “operations” of the complement system has provided crucial insight into how its delicate equilibrium is maintained, hence ensuring a robust state of homeostasis. However, external factors, particularly lifestyle choices, such as smoking, can significantly disrupt this balance, leading to aberrant complement activation and contributing to the pathogenesis of various conditions. In the next section we will delve into the specific impact of smoking on the complement system and its different components in the context of diseases states. We will also highlight the pathophysiological consequences and the underlying mechanisms through which smoking exacerbates disease progression via complement system dysregulation.

## Smoking-induced dysregulation of the complement system: pathways to pathology

The impact of smoking on complement activation was explored very early in a study that examined its effects on serum complement using Alternative Complement pathway hemolytic activity (ACH50) assay. This work revealed that the alternative complement pathway is activated, particularly in chronic elderly smokers (42) but this work did not explore this relationship in the context of disease conditions. Nonetheless, separate studies explored the complex interplay between smoking and the complement system across various health contexts, underscoring the significant impact of cigarette smoke on cardiovascular, respiratory, and immune system functions, as well as its role in the parthenogenesis of diseases that impact these systems, including development of cancer. Collectively, these studies showcase the critical role of complement activation in the pathophysiology of smoke-induced damage and disease.

### Complement activation and smoking induced cardiovascular diseases

The activation of complement components has been shown to predict adverse cardiovascular events, such as myocardial infarction (43). Furthermore, complement factors such as C3 and C4 were found to be linked to the presence of atherosclerosis (44, 45). In fact, the deposition of the complement components C1q, C3, and C4 and generation of the terminal complement complex C5b-9 in atherosclerotic lesions was unequivocally illustrated (by immunohistochemistry) (46). There is also evidence that in patients with type 2 diabetes who have experienced myocardial infarction (MI), elevated levels of soluble sC5b-9 can be/are indicative of an increased risk for future cardiovascular events (47). This suggests that the complement system is significantly involved/plays a major role in the development of cardiovascular disorders. Therefore, exploring complement activation triggers such as smoking offers opportunities to enhance our understanding of the pathophysiology of the aforementioned diseases.

The association between plasma complement C3 levels and coronary heart disease (CHD) within the context of smoking behavior was examined in the CODAM (Cohort on Diabetes and Atherosclerosis Maastricht; sample size n= 562) study (48). This study revealed a significant interaction between high plasma C3 levels and heavy smoking in relation to the prevalence of CHD. Specifically, while no substantial association between C3 levels and CHD prevalence was observed among never and light smokers, a strong association emerged in those who are heavy smokers. This association persisted even after adjustments for traditional cardiovascular risk factors, including the metabolic syndrome, C-reactive protein levels, and insulin resistance; which clearly indicates that the relationship between high C3 levels and CHD in heavy smokers is independent of these factors (48). Furthermore, separate reports suggested that complement activation, specifically, elevated levels of C3a and C5a are linked to cardiovascular complications and adverse cardiovascular events, respectively (49, 50).

In a different but related context, another study investigated the impact of tobacco (smoke and extract) and shear stress on complement activation in human endothelial cells (51). The results showed that tobacco smoke extract and shear stress both enhanced the deposition of the complement component C4d on endothelial cells, but did not significantly affect the deposition of C3b or C5b-9, suggesting limited activation of the complement system. This work also revealed and in a similar manner to tobacco extract, that the combination of tobacco smoke and shear stress significantly increases C4d deposition, confirming a synergistic effect on complement activation. Importantly, despite this activation, increases in C3b or C5b-9 were not observed similar to smoke extract. Accordingly, this regulation could be attributed to the concurrent increase in the expression of complement regulatory proteins CD35 (CR1) and CD55 on endothelial cells that are exposed to both stressors (52). These findings together suggest a mechanism to prevent excessive complement activation and potential vascular injury. The same research team conducted a similar set of experiments using endothelial cell lines (HUVEC) and human bone marrow microvascular endothelial cells (BMEC) and showed increased C4d deposition after treatment with tobacco smoke or exposure to shear stress, whereas their combined effects resulted in a two-fold increase in C4d deposition (53). This was accompanied by a 50% increase in surface gC1qR/p33 receptors and a 33% increase in ICAM-1 expression (53). These studies indicate that the interplay between smoking, sheer stress, and complement system in inducing endothelial activation is complex. This complex relationship might also hint that the impact of smoking and sheer stress on the complement system in the context of cardiovascular disease could be moderated/mediated by other unknown sheer stress variables such as blood viscosity, internal diameter, physical dimension of the blood vessels, and blood velocity as well as unknown smoking variables such as the specific chemical composition and concentration; which are areas that require more examination.

The decline in traditional smoking seems to have been associated with increased prevalence of novel tobacco products such as electronic/e-cigarettes, in part because of misconceptions regarding their safety (54). The activation of complement components was also illustrated in the context of endothelial cells in response to e-cigarettes (vapor) exposure. To this end, one study showed a significant increase in complement deposition and expression of the receptors for C1q. Interestingly, these results were independent of nicotine and the exposure to e-vapor was just as harmful as tobacco smoke extracts (55). E-cigarette role in activating complement components was also assessed *in vitro* in immortalized Kupffer cells, to determine their role as promoters of cardiovascular diseases through propagating the state of inflammation (56). The study did show- after exposure to e-cigarette extracts- an increase in the deposition of complement products C1q, C3b, C4d and C5b-9 on Kupffer cells, that also coincided with increased surface expression of globular head of C1q (gC1qR) and receptor for the collagen region of C1q (cC1qR) (56).

More recently, a study that examined the impact of e-cigarette extracts on platelets- which are major players in development of cardiovascular disease (57)) (58)- and as expected, showed enhanced function, including platelet aggregation, adhesion and secretion. Interestingly, it was observed that exposure of platelets to e-cigarette extracts induced significant up-regulation in the expression of the platelet receptor for the globular head of C1q (gC1qR) and receptor for the collagen region of C1q (cC1qR). Furthermore, a marked increase in the deposition of C3b and C4d was also observed; which was inhibited by pure nicotine, suggesting an antagonistic role (58). These data highlight the complexity of the interaction between complement components, their target cells and the state of the surrounding environment. However, whether such impact of e-cigarettes on the complement can actually lead to the development of pathology clearly remains an area that requires further investigation.

It is noteworthy that a study evaluated the combined effects of aging and cigarette smoking on stroke outcomes and the potential benefits of anti-complement mitigation strategies, in mice. The study used B4Crry, a single-chain variable fragment (scFv) antibody derived from the B4 IgM monoclonal antibody (a self-reactive pathogenic natural IgM antibody). B4Crry is linked to murine complement receptor 1 (CR1)-related gene/protein y (CRRY), an ortholog of the human complement receptor 1 (CR1), and serves as a targeted complement inhibitor that blocks all complement pathways at the central C3 activation step (59). This study did highlight the exacerbated inflammatory effects of aging and smoking on stroke outcomes, which were significantly mitigated by complement inhibition, thereby underscoring the critical role the complement system plays in the pathophysiology of stroke, including in the context of smoking (60).

Finally, another study investigated the interaction between smoking and a complement gene polymorphism (C4B*Q0, a silent allele of the C4B gene) in promoting cardiovascular disease morbidity and mortality (61). It was found that among smokers, carriers of the C4B*Q0 allele had a significantly higher frequency of cardiovascular disease compared to non-carriers and non-smoking subjects. The authors hypothesized that low levels of C4B can lead to an impaired ability to eliminate immune complexes (that is, the removal of immune aggregates through erythrocyte complement receptor CR1) (61, 62). Indeed, a less efficient and reduced ability to tag and clear immune complexes (aggregates of antigens and antibodies) can lead to accumulation of the immune complexes in tissues and the bloodstream, leading to chronic inflammation and potential tissue damage (26). Thus, this work underscores the impact of the interaction between genetic predisposition and smoking in the development of cardiovascular diseases, and emphasizes the importance of identifying genetic risk factors that may enhance the detrimental effects of smoking on cardiovascular health (61).

Collectively, the aforementioned studies/findings illustrate the complex role of complement activation and its interplay with environmental factors such as smoking, and highlight the delicate balance between activation and regulation and how that influences the progression of disease.

### Complement activation and smoking induced respiratory diseases

Studies have suggested that the complement system plays a multifaceted role in respiratory system defense mechanisms, and hence could potentially contribute to the development of pathological processes. For instance, activation of complement pathways can lead to severe respiratory inflammation and tissue damage (63, 64). Furthermore, complement activation is also thought to participate in progression of various chronic lung diseases, including cancer (65–68). In light of this notion, exploring the impact of smoking on complement activation as a mechanism of lung disease development is warranted. To this end, the role of C1q, a complement protein 1 complex component, in the development of emphysema through cigarette smoke exposure was examined (69). This work revealed that chronic cigarette smoke exposure downregulated C1q in lung antigen-presenting cells in both humans and mice. This downregulation was associated with enhanced lung inflammation, increased Th17, which is a subset of CD4+ T helper cells, and the promotion of emphysema, suggesting that cigarette smoke-induced reduction of C1q contributes significantly to the loss of peripheral tolerance. This occurs when the mechanisms that normally keep self-reactive immune cells “in check” fail, thereby allowing them to get activated and attack the body’s own tissues (70), which leads to emphysema (69).

Another study conducted in the context of investigating the mechanism of complement-induced lung emphysema demonstrated that cigarette smoke extract has the capacity to modify the complement (C3) and activate the alternative pathway, *in vitro (* 71). This work detailed that cigarette smoke activates the alternative pathway of complement by specifically modifying C3 and that these modifications include cleavage of its thiolester bond and formation of C3 multimers. Together, these findings elucidate the molecular mechanism by which cigarette smoke activates the complement system and provide insight into how that potentially contributes to the inflammatory processes observed in the lungs of smokers (71). Along the same lines, it was demonstrated that exposure to cigarette smoke-dependent activation of the complement system results in increased neutrophil and monocyte chemotactic activity. This was evidenced by the significant attraction of neutrophils and monocytes to serum exposed to cigarette smoke. The increase in chemotactic activity was partially attenuated by EDTA, suggesting activation of the alternate complement pathway, which does not require the presence of antibodies. Further analysis revealed cleavage of properdin factor B and C3 in smoke-exposed serum, along with detection of C5a, which indicates complement activation (72). These findings suggest that complement activation by cigarette smoke may play a role in directing the influx of neutrophils and monocytes into the lungs of smokers, and contribute to the pathogenesis of smoking-induced lung diseases. In this connection, a separate study investigated the effects of smoking on peripheral leukocyte counts and serum components related to leukocytes, such as complement in smokers without lung diseases, and showed contrasting findings. Thus, it was found that peripheral leukocyte numbers increased with the duration and quantity of smoking, independent of age or gender, however though, serum levels of complement (CH50 and C3a) did not significantly change with smoking. Additionally, there was no correlation between neutrophil counts in the peripheral blood and bronchoalveolar lavage fluid, indicating that the increased leukocyte numbers in smokers might not be due to complement activation (73). While these contrasting results can be attributed to differences in methodology and experimental settings, they do nonetheless highlight the need for more careful examination of the link between smoking and complement activation in the context of lung diseases, under different real-life experimental settings.

Complement components were also investigated as biomarkers of lung malignancy. To this end, a study highlighted that lung tumor cells, compared to non-malignant bronchial epithelial cells, activate the classical complement pathway more robustly via direct binding of C1q, leading to the generation of elevated levels of C4d, a stable degradation product of complement activation. This elevation in C4d is consistently observed in lung tumor tissues, bronchoalveolar lavage fluid, and plasma of lung cancer patients, and correlates with poor prognosis (74). Importantly, these increases are not observed in smoking-related inflammatory lung conditions such as emphysema or COPD, emphasizing the specificity of C4d as a biomarker for malignancy rather than for smoking-induced inflammation. Interestingly, despite heightened complement activation, lung tumor cells evade complement-mediated cytotoxicity by expressing and binding complement factor H (CFH), a key regulatory protein that inhibits the alternative pathway of complement activation (75, 76). This allows tumor cells to harness the immunomodulatory effects of complement activation—such as inflammation and microenvironment remodeling—while avoiding its destructive consequences. Together, these findings reveal a unique tumor-associated complement activation signature in the lung, with potential utility in distinguishing malignant from non-malignant smoking-related lung pathologies as well as for informing prognosis.

Evidence indicates that smoking is the most important factor for developing lung cancer (77). To this end, and consequently, the interaction between cigarette smoking and the complement components such as factor H (CFH) variant Y402H (rs1061170) in relation to lung cancer risk was examined. In this study that included 1000 lung cancer cases and 1000 controls (Chinese population), it was found that the CFH Y402H genotypes were significantly overrepresented among lung cancer patients compared to controls, with the 402His/His or 402His/Try genotypes associated with a higher risk of lung cancer (OR = 1.50, 95% CI = 1.12–2.00). Importantly, the increased cancer risk associated with CFH variants was evident among smokers but not non-smokers, suggesting a smoking-related genetic risk factor for lung cancer. Further analysis showed that the risk increased with higher cumulative smoking doses, which suggests that the CFH Y402H polymorphism may interact with cigarette smoking and amplify lung cancer development risk in the Chinese population (78).

Collectively, these studies highlight the intricate interplay between the different complement system components and smoking in modulating the risk of lung diseases, including lung cancer. These findings also further underscore the importance of considering both genetic susceptibility and lifestyle factors in understanding and potentially mitigating disease risk.

### Complement activation, smoking, inflammatory mucosal and macular degeneration diseases)

As indicated before, the activation of the complement system is a significant factor in the development and exacerbation of inflammation (10, 79), and it plays a multifaceted role in various inflammatory disorders (80, 81), contributing to tissue damage (82) and disease progression (83). Similarly, smoking is intricately linked to inflammation through various mechanisms, such as oxidative stress (84), alteration of immune responses (85, 86), which supports its capacity in inducing chronic inflammation (87), all of which can contribute to the development of a range of diseases. Given this intertwined relationship between smoking, inflammation, and complement activation, it is imperative to highlight this notion, in an attempt to offer a better understanding of the potential mechanisms that can lead to the genesis of disease. To this end, *in vitro* experiments demonstrated that exposure of human respiratory epithelial cells to cigarette smoke extract resulted in complement activation, as evidenced by increased levels of complement activation fragments (C4a, C3a, and C5a) and immunofluorescent staining for C3d (88). Moreover, an *in vivo* study using complement-deficient (*C*3^-^/^-^) mice exposed to cigarette smoke showed a significant reduction in nasal damage. This suggests that cigarette smoke activates the complement system, and contributes to mucosal damage, and that complement deficiency provides a protective effect against smoke-induced nasal injury (88). Similarly, it was demonstrated that extracts from various smokeless tobacco products, including loose leaf chewing tobacco, dry snuff, and moist snuff, depleted the complement hemolytic activity in normal human serum, and that they did so in a dose-dependent manner (89). This complement depletion was largely due to the consumption of C3, a key component in the complement cascade. The presence of significantly elevated levels of complement cleavage fragments iC3b, Bb, and- particularly with moist snuff- C4d, indicates activation of both the alternative and classical pathways. This complement activation by smokeless tobacco extracts suggests a potential mechanism for initiating inflammation of the oral mucosa in users of these products (89).

With regard to macular degeneration, it has been shown that chronic smoke exposure leads to ocular damage due to involvement of the alternative complement pathway. This was demonstrated by the finding that deficiency of alternative complement pathway (using *CFB* ^-^/^-^ mice) can ameliorate age-related macular degeneration (AMD) associated with smoking (90). In a similar context, retinal pigment epithelium (RPE) cells exposed to smoke revealed AMD through the process of oxidative stress and alternative complement pathway activation. This activation is linked to endoplasmic reticulum stress and subsequent lipid accumulation within the RPE, highlighting a synergistic role of oxidative stress and complement activation in AMD pathogenesis. Furthermore, using antioxidants and inhibitors to block the alternative complement pathway signaling led to mitigation of smoke-induced damage in the RPE and prevented or slowed AMD progression (12). Additionally, the relationship between smoking and complement activation in inducing AMD was also shown to be dependent on the translocation of antioxidant transcription factor nuclear factor erythroid 2-related factor 2 (Nrf2) in RPE cells, which led to an increase in C3a and C3b as well as a decrease in complement regulators such as CD46, CD55, and CD59. Additionally, Nrf2 knockdown amplified cigarette smoking-induced increases in C3a and C3b, indicating that Nrf2 might- at least in part- play a protective role against cigarette smoking-induced pro-inflammatory responses in RPE cells (11). Another important aspect in the pathogenesis of AMD was related to a genetic risk linked to the region spanning from the CFH gene to the F13B gene on chromosome one. The data supported the notion that smoking was associated with increased terminal complement component and C-reactive protein levels in various parts of the macula, indicating elevated levels of complement activation and inflammation associated with both genetic risk at the CFH-to-F13B locus and cigarette smoking (91).

### Complement–coagulation interplay under smoke and vape exposure

Evidence has demonstrated that the complement and coagulation systems/cascades do not operate in isolation; rather they exhibit significant cross-talk (31). For example, thrombin can generate the complement fragment C5a even in the absence of the usual complement convertases (32). Likewise, coagulation factor XIIa, kallikrein, and plasmin can cleave complement proteins to produce active fragments (92). Reciprocally, complement activation produces inflammatory mediators (C3a, C5a) that can enhance the pro-coagulant activity on cell surfaces, thereby promoting clot formation. This bidirectional “liaison” means that an insult activating one system often propagates a cascade in the other, creating a “thromboinflammatory” response (93). In healthy conditions, regulatory mechanisms keep both systems in check, but environmental stressors like tobacco smoke or other forms of environmental exposure can destabilize this balance toward pathological activation. To this end, we highlighted evidence of smoking/vaping-induced complement activation, which participates in disease development. Similarly, Smokers exhibit higher circulating levels of coagulation/clotting biomarkers, such as fibrinogen, D-dimer (fibrin degradation product), and homocysteine, reflecting a tendency toward hypercoagulation (94). Additionally, when the complement and coagulation systems are co-activated by smoking or vaping, they can amplify each other’s effects. For example, complement C5a produced in smoke-exposed tissues can stimulate neutrophils and monocytes to release tissue factor-rich microvesicles, which accelerates coagulation. Conversely, thrombin generated in smokers may further cleave complement components, thereby creating a vicious cycle. A recent proteomic study in a mouse smoking model illustrates this interplay: cigarette smoke led to a prothrombotic shift in lung lymphatic fluid with concurrent upregulation of coagulation and multiple complement proteins (95). These findings clearly underscore the notion that smoking can simultaneously engage the complement cascade and the clotting cascade, creating a compounded inflammatory-coagulatory response. Nevertheless, more studies will be needed to solidify this relationship in the context of chronic diseases development in both smokers and vapers, and determine the possible toxic ingredient(s) that are responsible for such an effect.

### Complement activation by smoking: possible suspects

Concerning the potential tobacco chemicals that might activate the complement system, while the data available thus far are limited, activation of the classical pathway by tobacco glycoprotein (TGP)- which is derived from tobacco leaves- and a similar substance from cigarette smoke condensate- referred to as TGP-S- was explored (96). The data showed that these substances activate the complement system through direct interaction with the C1q component, which was indicative of a mechanism for classical pathway activation. A key finding is that TGP and TGP-S, by binding to C1q, can initiate complement activation, albeit TGP-S does not lead to the formation of a C3 cleaving enzyme, suggesting only partial activation. Additionally, the study provides evidence that polyphenols associated with tobacco, such as chlorogenic acid and rutin, can mimic the activation effects of TGP and TGP-S, further implicating/highlighting a role for tobacco’s chemical constituents in complement activation (96). Consistent with the TGP effect on immunity, other studies have also shown that TGP is in fact capable of altering the immune system, through stimulation of B cell and T cell proliferation (97). However, this notion (TGP effects) was challenged by others, as it was shown that it is the TGP isolation protocol (98) that yielded contaminants- mainly polyacrylate- that are responsible for this activity; rather than TGP itself. Nevertheles, this area of research is understudied, and more work needs to be done to clearly understand/uncover the role of TGP in modulating the complement system (99).

Smoldering tobacco products produce particulate matter (PM), which is a major indoor air pollutant that has been linked to multiple diseases, including cancer (100–102). To this end, PM, which is a mixture of different-sized liquid and solid particles, varies in source and composition (103). Traditionally, PM is classified according to particle size, which correlates with the penetration into the respiratory tract. The smaller the particles, the deeper they penetrate and the more severe the associated health effects are (104, 105). Consequently, PM could be another suspect that can activate the complement system. Indeed, there is data showing that the development of airway hyperresponsiveness (AHE) is a complement-mediated process (20). In this connection, PM exposure of wild-type mice resulted in significant increases in AHR, whereas it did not significantly increase airway reactivity in (*C*3^-^/^-^) mice (20). Similarly, it was also shown that PM is capable of activating the complement system as a part of a “pan-activation” that also involves coagulation and the Kallikrein-Kinin systems. This specific activation was observed both *ex vivo* and *in vivo* through the rise in both C3a and C5a, which was found to be mainly mediated by fine particulate matter *PM*_2.5_ (106). PM was also shown to activate complement through membrane attack complex (MAC) on the surface of endothelial cells, which led to the promotion of inflammation (107).

Tobacco smoking was also linked to a profile of toxic metals such as arsenic (As), cadmium (Cd), chromium (Cr), nickel (Ni), and lead (Pb) (108). These metals were shown to be associated with negative health effects, including cancer (109), cardiovascular, and renal diseases as well as other health issues (110, 111). Thus, the association between exposure to metals such as copper, zinc, and arsenic, and serum levels of the complement components C3 and C4 was explored (112). In a recent study (with a sample size of (n=2,977)), 17 plasma metals, serum C3 and C4 levels, and genetic risk scores (GRSs) derived from single nucleotide polymorphisms that are associated with C3 or C4, were investigated. A positive association between plasma copper levels and both C3 and C4 levels, and between plasma zinc levels and C3 levels was found. Additionally, a significant interaction was observed between arsenic exposure and C3 genetic predisposition in relation to serum C3 level, suggesting that arsenic exposure could modify the association between genetic predisposition to C3 levels and actual serum C3 levels (112). Collectively, data from this study provides insight regarding the impact of metal exposure on the human immune system, particularly the complement system.

**Figure 2:**
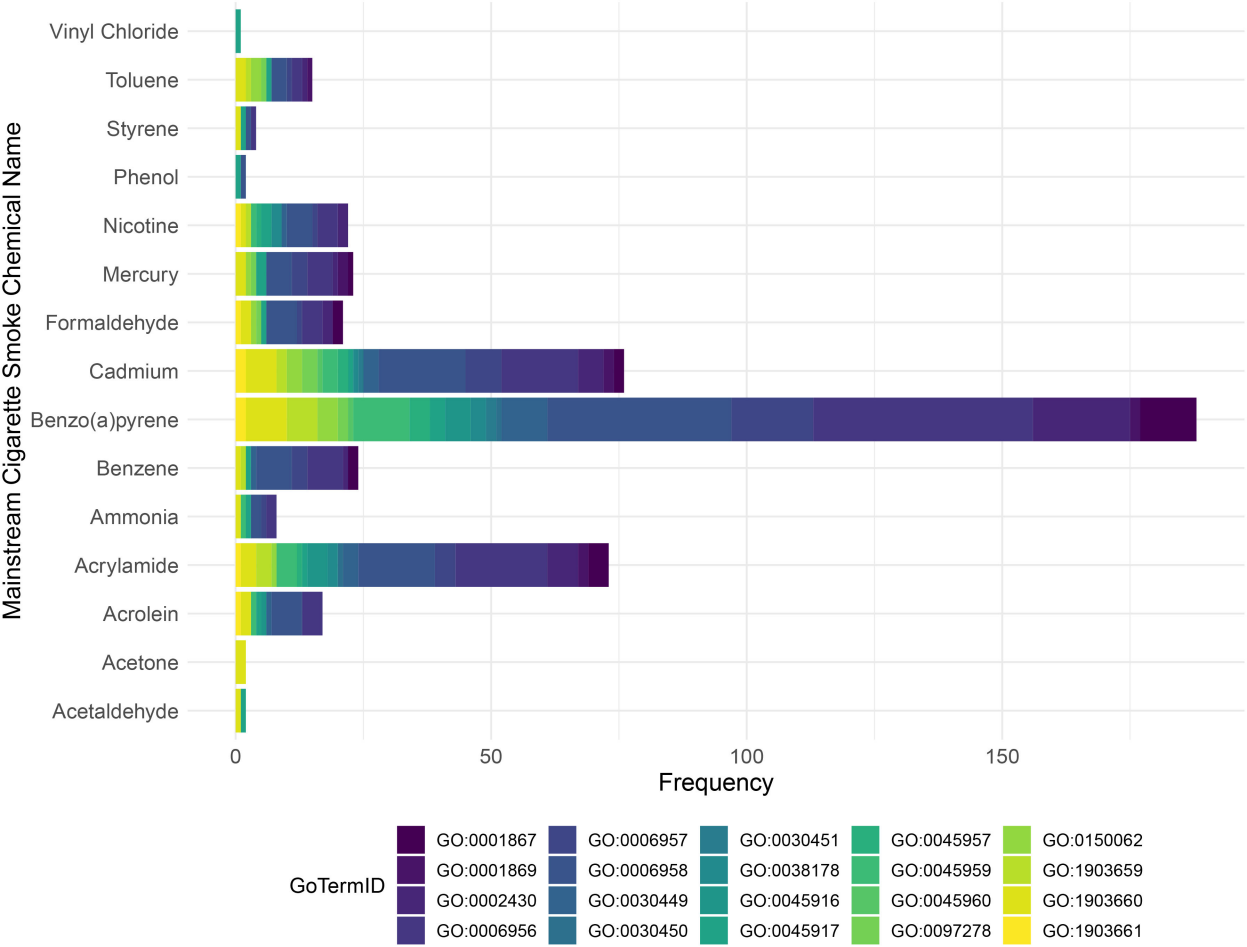
Stacked bar chart illustrates the distribution of Gene Ontology (GO) terms for each mainstream tobacco chemical according to the Comparative Toxicogenomics Database (CTD). The bar chart displays the frequency of GO terms for each chemical, with the bars stacked and colored according to different GO term IDs. The mainstream smoke tobacco chemicals are listed along the y-axis, and the frequency of each GO term is shown on the x-axis.

As was highlighted earlier in this review, tobacco smoking is linked/contains a host of different chemicals, some of which are known to be highly toxic (113). In this connection, the toxic profile of tobacco mainstream smoke was characterized in different studies (114, 115), which makes it possible to investigate the impact of some of these chemicals on the complement system, using curated databases such as the comparative toxicogenomic database (CTD) to delineate possible impacted pathways (116). Hence, we first used these studies to extract the mainstream cigarette smoke chemical profile (list of chemicals “produced” by mainstream cigarette smoke). We next utilized the CTD to enrich for complement biological processes to determine if there are any interactions between these chemicals and the complement system (**Figure 2**). Our analysis highlights the different complement processes affected by each mainstream cigarette smoke chemical, with Cadmium and Benzo(a)pyrene showing notably high frequencies of association with complement gene ontology (GO). Specifically, complement activation (GO:0006956) showed highest frequency in benzo(a)pyrene, acrylamide, and cadmium (**Figure 2**). Benzo(a)pyrene also showed high frequency for complement receptor mediated signaling pathway (GO:0002430) as well as complement activation-alternative pathway (GO:0006957) (**Figure 2**). These data- although predictive in nature- do provide new and important insight as to the possible culprit chemicals of tobacco smoke that might be engaged in activating the complement system and participating in the development of various disease states.

Taken together, these data provide evidence supporting the notion that tobacco smoking- through its constituents- can trigger activation of the complement system, which in turn participates in initiating an inflammatory state and creates/contributes to a host of disease conditions.

## Conclusion

This review underscores the profound and multifaceted impact of smoking on the complement system and highlights the intricate mechanisms through which tobacco use exacerbates the pathogenesis of a wide range of diseases. The evidence presented highlights the dual nature of the complement system—on one hand, it serves as a critical player/defender against invaders, whereas on the other hand, it appears to also be a potential perpetrator of tissue damage, inflammation, and diseases when dysregulated by external factors such as smoking. Through detailed exploration of the literature, we have delineated how smoking influences complement activation, and contributes to the genesis of cardiovascular, respiratory, and a host of inflammatory and degenerative conditions.

Crucially, the interaction between smoking and the complement system is not merely a linear process, but rather involves a complex network of genetic, biochemical, and environmental factors. To this end, genetic predispositions, such as polymorphisms in complement-related genes, can significantly modulate the impact of smoking on disease risk and progression, emphasizing the importance of a personalized approach in understanding and managing these risks. Moreover, through work by us and others, the specific components of tobacco smoke, including chemical constituents like TGP and PM, as well as toxic metals, and potentially other mainstream smoking chemicals have been identified as key players in complement activation. The implications of these findings are at least two folds: firstly, they provide a compelling argument reaffirming the need for public health initiatives aimed at reducing tobacco and tobacco-related products use, given the clear link between smoking and disease development; and secondly, they highlight the urgent need for further research into targeted therapies that can mitigate the effects of complement activation in smokers, while also potentially offering new avenues for the treatment of smoking-related diseases. This can be achieved, for example, by targeting specific components of the complement system, such as anaphylatoxins (C5a and C3a) and their receptors that are known for their potent inflammatory effect. However, more work is needed in order to be able to fine-tune complement activation using a targeted approach. In conclusion, this review not only adds to the body of evidence on the harmful effects of smoking, but also-importantly- opens new research directions for (mechanistic) understanding of the complex interactions between smoking, the complement system, and the pathogenesis of a host of diseases. It also calls for a holistic research approach that combines public health measures, individual genetic risk assessment, and innovative therapeutic strategies to combat the global health crisis posed by tobacco use.
